# Striatal Dysregulation of *Angpt2* and Circadian Gene Expression in a Rotenone Rat Model of Parkinson’s Disease

**DOI:** 10.1007/s12031-026-02506-z

**Published:** 2026-04-02

**Authors:** Dalia Y Al Saeedy, Elisa Hawkins, Mikhail G Dozmorov, Ohm Tripathi, Sina Mahdiani, Fay M Jahr, Ola AlAzzeh, Laxmikant S. Deshpande, Joseph L. McClay

**Affiliations:** 1 Department of Pharmacotherapy and Outcomes Science, School of Pharmacy, Virginia Commonwealth University, Richmond, VA USA; 2 Department of Neurology, School of Medicine, Virginia Commonwealth University, Richmond, VA USA; 3 Department of Biostatistics, School of Public Health, Virginia Commonwealth University, Richmond, VA USA; 4 Department of Pharmacology and Toxicology, School of Medicine, Virginia Commonwealth University, Richmond, VA USA; 5 Virginia Institute for Psychiatric and Behavioral Genetics, Virginia Commonwealth University, Richmond, VA USA

**Keywords:** Gene expression, RNA sequencing, Circadian rhythms, Pesticides, Neurodegeneration

## Abstract

Rotenone is a naturally-occurring isoflavone that is used as a pesticide. Rotenone is also administered to rats to induce nigrostriatal dopaminergic neuron loss in an established model of Parkinson’s Disease (PD). However, the molecular mechanisms linking rotenone action to the emergence of PD-like phenotypes are poorly understood. Here, we characterize rotenone-induced gene dysregulation in the striatum. Male Lewis rats at 12–14 months received rotenone injected at 3 mg/kg, i.p. once daily for nine days. Behavioral effects of rotenone were verified using the bar test for catalepsy. RNA sequencing was carried out on RNA extracted from the striatum of rats receiving the full course of Rotenone treatment and vehicle-treated controls. Illumina PE150 sequencing to 30 M clusters per sample revealed several hundred differentially expressed genes (DEGs) at FDR < 5%. These included Dopa decarboxylase (*Ddc*), which encodes an important enzyme in dopamine production, and Angiopoietin 2 (*Angpt2*), a gene previously implicated in analysis of post-mortem PD brain. Pathway analysis of top findings identified the Circadian Clock System as enriched with rotenone DEGs. Circadian and sleep dysfunction is a known feature of PD. We validated the differential expression of two circadian genes via quantitative PCR: downregulation of Period 3 (*Per3*) and upregulation of the aryl hydrocarbon receptor nuclear translocator-like (*Arntl*). Overall this study represents a first look at striatal dysregulation of gene expression in the established rotenone PD model and indicates that further study of circadian gene dysregulation in this model may be fruitful.

**Supplementary Information:**

The online version contains supplementary material available at 10.1007/s12031-026-02506-z.

## Introduction

Parkinson’s disease (PD) is a chronic and progressive neurodegenerative movement disorder with clinical manifestations such as muscle stiffness, rigidity, resting tremors, bradykinesia, gait disturbances, postural instability and alterations in speech. Fundamental pathological features of PD include the progressive loss of nigrostriatal dopaminergic neurons and peripheral autonomic neurons, in addition to the presence of Lewy bodies, which are accumulations primarily composed of α-synuclein protein, within neurons (Morris et al. 2024). By 2016, PD was estimated to affect over 6 million individuals globally, more than double the estimated number in 1990 (2.5 million). This trend is expected to persist due to rising life expectancy and global population growth (Deliz et al. 2024). The increase in prevalence of PD can be linked to various factors, including advancements in detection and diagnosis methods, increased awareness of the condition, extended life expectancy, and potentially elevated environmental toxin exposures, such as those associated with agriculture and industrialization, i.e. pesticides, solvents, and metals (Armstrong and Okun 2020). Genetics also plays a role and it is estimated that about 5–10% of PD cases are attributable to mutations in known PD risk genes, which causes monogenic or idiopathic PD (Ohnmacht et al. 2020; Bloem et al. 2021). Genome-wide association studies have mapped close to one hundred risk loci for non-monogenic or sporadic PD (Nalls et al. 2019; Kim et al. 2024). The reported heritability of PD varies somewhat across studies, but most estimates are in the range of 20–30% (Billingsley et al. 2018; Nalls et al. 2019; Ohnmacht et al. 2020; Bloem et al. 2021; Kim et al. 2024). Therefore, even though PD genetics is highly complex, clearly a substantial portion of PD risk is attributable to non-genetic factors.

Shedding light on environmental risk factors for PD presents a challenge, but there is compelling epidemiological evidence that agricultural workers exposed to certain pesticides, such as paraquat, rotenone, and chlorpyrifos, are at an increased risk of developing PD (Brown et al. 2006; Ascherio et al. 2006; Narayan et al. 2013). In addition, 1-methyl-4-phenyl-1,2,3,6-tetrahydropyridine (MPTP) has previously been identified as a causative agent of striatal dopamine loss in young drug abusers, leading to severe rapid-onset treatment-resistant Parkinsonism (Nonnekes et al. 2018). Although MPTP is not commonly encountered in the environment, it provides further evidence that certain toxin exposures can lead to cellular dysfunction that causes PD.

The mechanistic link between toxin exposure and the development of PD has been extensively investigated. Several rodent neurotoxic models of PD have been established that show significant loss of nigrostriatal dopaminergic neurons, including those using substances such as 6-hydroxydopamine (6-OHDA), MPTP and paraquat (Bové and Perier 2012; Imbriani et al. 2022). Also among these established models is the rat rotenone model of PD, introduced by (Betarbet et al. 2000). Rotenone is a phytogenic flavonoid used as a pesticide, which can easily penetrate the blood-brain barrier due to its highly lipophilic nature. Once it enters neurons, it inhibits mitochondrial complex I leading to extensive production of reactive oxygen species (ROS) and impairment of proteasome function, causing proteolytic stress. The rat rotenone model results in the selective loss of nigrostriatal dopaminergic neurons and, notably, the formation of Lewy body-like inclusions containing α-synuclein. Behavioral symptoms include reduced movement, stooped posture, and significant stiffness, with similar effects seen through subcutaneous or intraperitoneal administration (Sherer et al. 2003; Höglinger et al. 2003; Bové and Perier 2012). The rotenone model is well-established and is considered a significant advancement in neurotoxic models of PD (Cannon et al. 2009; Tanner et al. 2011). However, the full extent of the mechanisms underlying rotenone-induced neurotoxicity leading to PD-like symptoms and pathology are not understood. Genomic technologies such as RNA sequencing (RNA-seq) are powerful tools to conduct exploratory analysis of genomic dysregulation, and to the best of our knowledge RNA-seq has not been used previously to study the effects of rotenone in the brain. Therefore, in the current study, we will use the rotenone rat model of PD to assess striatal dysregulation of gene expression induced by rotenone, to determine potential overlap with known risk genes and pathways in PD.

## Methods

### Rotenone Preparation

Rotenone was prepared as a 50× stock in 100% dimethylsulfoxide (DMSO) and diluted in medium-chain triglyceride Miglyol 812 N to obtain a final concentration of 3.0 mg/mL rotenone in 98% Miglyol 812 N, 2% DMSO (Cannon et al. 2009). Vortexing this solution creates a stable emulsion. The solution was made fresh daily and gently vortexed before drawing each injection to prevent settling of rotenone causing accidental overdose.

### Subjects and Treatment

Our methods for rotenone administration follow those outlined by Cannon et al. (2009). Their model of daily intraperitoneal rotenone administration produces reproducible lesions of the nigrostriatal dopamine system associated with α-synuclein pathology and development of a PD-like phenotype. Forty male Lewis rats aged 12–14 months (middle-aged) were obtained from Hilltop, Scottdale, PA. Middle-aged subjects were chosen to mirror the age-dependent pathology of PD. They are also more sensitive to rotenone than younger rats and show less variability from starting rotenone treatment to onset of the PD-like phenotype (Cannon et al. 2009). Rats were acclimated inside our vivarium for one week before the injections began and housed at 20–22 °C with *ad libitum* access to standard rodent food pellets and water.

**Fig. 1:**
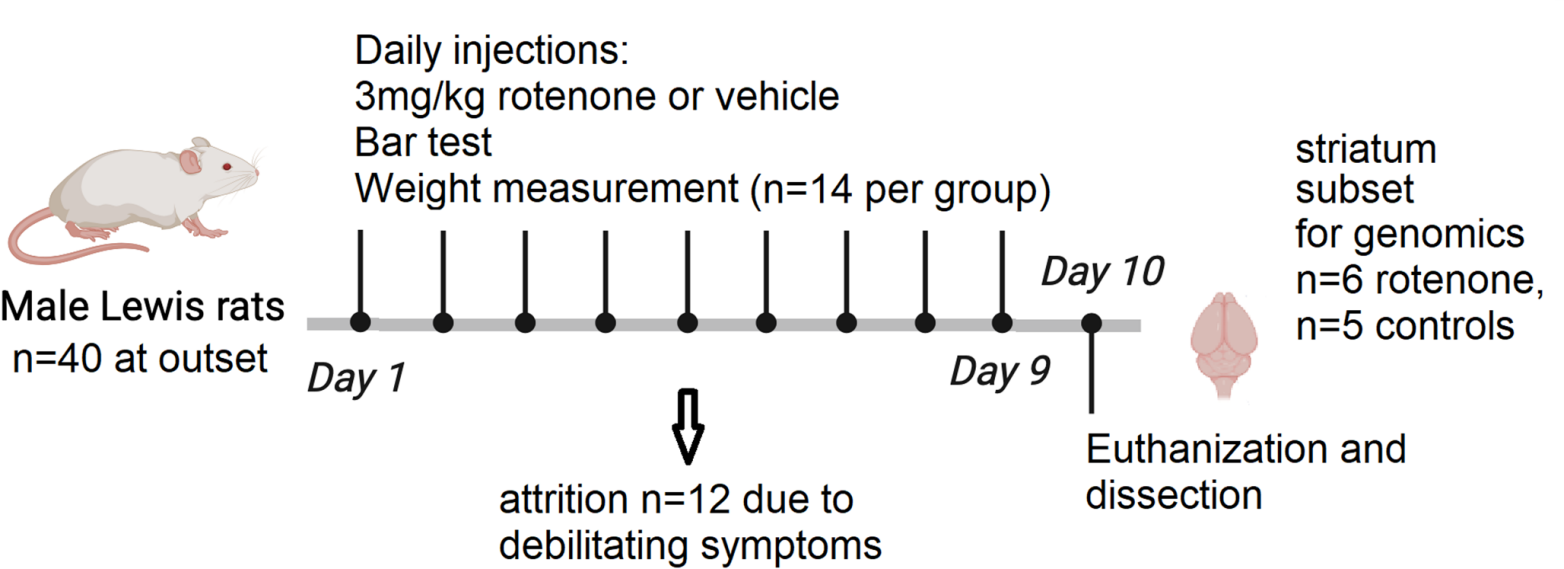
Experimental timeline of rotenone treatment. Rats received either rotenone (3 mg/kg) or vehicle for 9 days. Rotenone caused severe rigidity in some subjects (*n* = 12), which were euthanized and did not form part of the study. RNA extracted from striatal tissue from a subset of subjects receiving the full course of injections (*n* = 6 rotenone, *n* = 5 controls) was analyzed in our genomics assays

Zeitgeber time (ZT) denotes time relative to lights-on (ZT0 = lights on at 06:00; ZT12 = lights off at 18:00). Samples were collected during a two-hour window between 10:00–12:00 (ZT4-6). Rotenone solution was administered intraperitoneally (i.p.) at 1 milliliter per kilogram of body weight (Cannon et al. 2009). The experimental group received 3 mg/kg rotenone i.p. injections once daily for nine days, while the control group received once daily vehicle injections only (see timeline in Fig. 1). Animals were weighed daily prior to injections. During the course of the experiment, some subjects (*n* = 12) exhibited severe debilitating rigidity and were unable to move between days 5–9. These rats were euthanized and not included in the study. Rats receiving the full complement of nine rotenone injections and exhibiting signs of postural instability, catalepsy and bradykinesia but not debilitating rigidity were included for downstream analysis.

### Assessment of PD-like Signs

Subjects were assessed each day for the emergence of a PD-like phenotype 2-h after the rotenone injection. The onset of catalepsy, or muscular rigidity and immobility, was assessed using the “bar test”. Here, the forepaws of rats were placed on a 3 cm diameter cylindrical bar which was elevated 9 cm from the ground. The time taken by a rat to descend and correct its posture was noted, with a cut-off time of 180s (Alam and Schmidt 2004). A longer latency time to descend is indicative of postural rigidity (catalepsy), a phenotype resembling PD-like signs in rats. Statistical analysis of behavior and weight loss phenotypes used two-tailed t-tests with significance threshold of *p* < 0.05. Shapiro-Wilk tests were used to assess potential deviation from normality. PD-like phenotype and weight are reported for the *n* = 14 subjects per group receiving the full complement of nine daily injections.

### Tissue Collection and Nucleic acid Extraction

Twenty-four hours after the last rotenone injection and behavioral assessment, a subset of rats (*n* = 7 per group) were deeply anesthetized using isoflurane, rapidly decapitated, and their brains were isolated. Using a rat brain matrix (Braintree Scientific, MA) and reference coordinates from Rat Brain Anatomic Atlas (Paxinos and Watson), cuts were made between channels 10 (at optic chiasm) and channel 6 (midway to optic chiasm). This 2 mm thick region included area spanning bregma interval − 1.4 to + 1.7 that contained striatum. About four to five 500-micron cuts were made in this region. Each section was then carefully micro-dissected on ice under a dissecting microscope to isolate striatal regions inclusive of caudate putamen, globus pallidus, and subtantia nigra. Striatal dissections were successful for *n* = 6 rats receiving a full course of Rotenone and *n* = 5 vehicle controls. The dissected regions were then pooled and immediately flash frozen using isopentane, transferred to a labeled Eppendorf tube and stored at -80 °C until utilized for nucleic acid extraction. DNA and RNA were extracted from striatal tissue sections by a column-based purification technique (Qiagen AllPrep Kit) according to the manufacturer’s instructions. RNA was stored at − 80 °C until used.

### RNA Sequencing (RNA-seq) and Differential Expression Analysis

Striatal RNA from rats receiving a full course of Rotenone treatment (*n* = 6) and vehicle controls (*n* = 5) was assessed using a TapeStation (Agilent). RNA quality was high with all samples having RNA integrity number (RIN) ≥ 9 (mean = 9.3). 1 µg aliquots were shipped to the service provider GeneWiz (Azenta, South Plainfield, NJ) for sequencing. Raw sequence data are available to download from the Sequence Read Archive with accession number PRJNA1310653 (https://www.ncbi.nlm.nih.gov/sra/PRJNA1310653). Following alignment to the Rn7 rat reference genome (Dobin et al. 2013), differentially expressed genes (Robinson et al. 2010) were called using a False Discovery Rate (FDR) threshold of 5% (Benjamini and Hochberg 1995). To identify common biological themes, top genes were entered into pathway analysis using the online resource at GeneOntology.org (Mi et al. 2017; Gene Ontology Consortium et al. 2023). Further details are provided in the Supplementary Materials.

### Quantitative PCR (qPCR) Analysis of Specific Genes

300ng of total RNA was reverse transcribed using the iScript kit (BioRad) according to manufacturer’s instructions. TaqMan^®^ gene expression assays were carried out in triplicate technical replicates of each assay (genes of interest and endogenous controls) on a QuantStudio 3 (Applied Biosystems). Predesigned assays from Thermofisher were as follows: *Angtp2* (Rn01756774_m1), *Arntl* (Rn00577590_m1), *Ddc* (Rn01401189_m1), *Cry1* (Rn01503063_m1), *Per3* (Rn00709499_m1). *Gapdh* (Rn01775763_g1) was used as the endogenous control. Each target gene assay and endogenous control assay was run in triplicate for each sample and a standard curve of serially diluted RNA was included on each plate to test for reaction accuracy and efficiency. Each plate was run at 50 °C for 2 min and 95 °C for 10 min, followed by 40 cycles of 95 °C for 15 s and 60 °C for 1 min. Initial analysis was conducted in the ThermoFisher cloud with subsequent analysis using the ΔΔCq method and plotting of results using R. Significant differences in expression were called using two-tailed t-tests at *p* < 0.05. Shapiro-Wilk tests were used to assess potential deviation from normality.

## Results

### Behavioral Effects of Rotenone and PD-like Signs in Rats

**Fig. 2:**
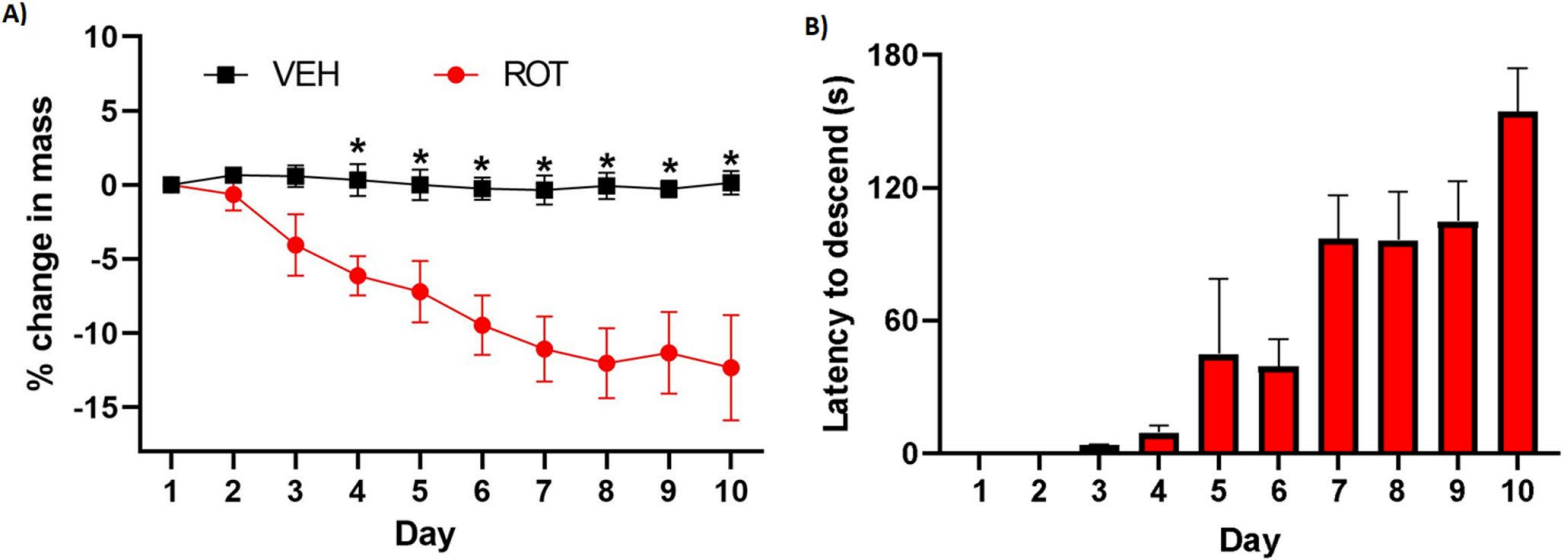
(**A**) Mean body weight after daily rotenone injections. Beginning day 5, daily i.p. rotenone elicited significant weight loss compared to the vehicle-treated group (*n* = 14/group, *p < 0.05). (**B**) Increased descend latency after daily rotenone injections. As the experiment progressed, rotenone-treated animals exhibited postural instability characterized by increased descent latency in the bar test. Data did not deviate significantly from normality using the Shapiro-Wilk test

Rotenone-treated animals were monitored daily for weight changes, mortality, and signs of PD-like phenotype. Rotenone-treatment was associated with progressive weight loss that approached approximately 10–15% compared to the vehicle-treated rats. Weight loss was significantly different between the two groups by day 4 of the experiment, and remained significantly lower in the rotenone-treated rats throughout the end of injection period (*n* = 14 per group, t-test, *p < 0.05, Fig. 2A).

The Bar Test was utilized to assess muscular rigidity in rats. The latency time in vehicle-treated rats was less than 2 s throughout the duration of the study. Rotenone-treated rats exhibited a latency time similar to the vehicle-treated rats for the initial four days. As shown in Fig. 2B, beginning the 5th injection, rotenone-treated animals exhibited postural rigidity characterized by a progressively increased descent latency in the bar test. The average descent latency for the rotenone-treated group approached the cut-off (maximum time) of 180 s by the end of the experiment (Fig. 2B), indicative of a significant rigidity and bradykinesia, signs commonly reported in PD.

### RNA Sequencing of Striatum

**Fig. 3:**
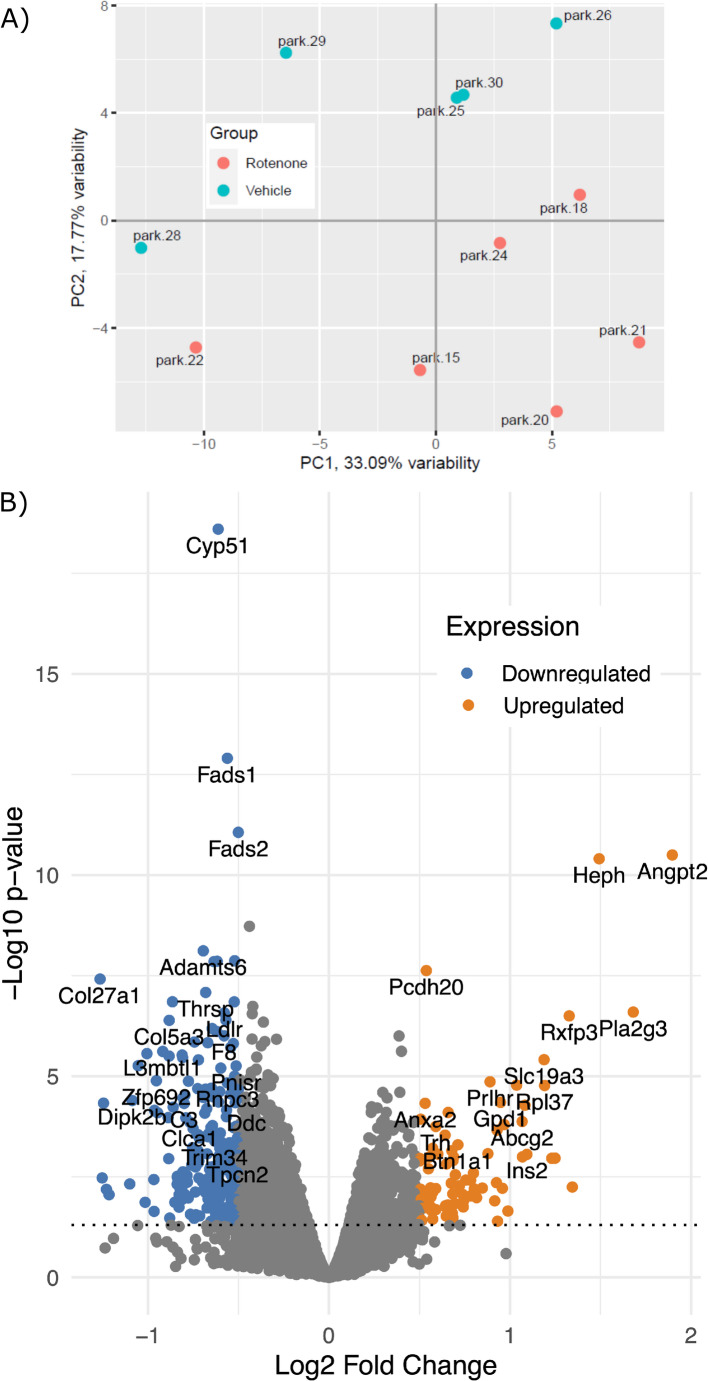
**(A)** Plot of sample loadings on the top two principal components extracted from the RNA-seq data. Overall the samples show heterogeneity but there is clear separation between the rotenone-exposed and vehicle control groups. **(B)** Volcano plot showing the top up-regulated and down-regulated genes. The y-axis is –log_10_(pvalue) while the x-axis is log_2_(fold change). The horizontal dotted line represents the threshold of genome-wide significance (FDR < 0.05). Samples are *n* = 6 rotenone-treated rats and *n* = 5 control rats

RNA was extracted from striatum from a subset of rats receiving the full nine-day course of rotenone administration (*n* = 6) and control subjects that received an equivalent number of vehicle injections (*n* = 5). RNA-seq was successfully conducted on these samples with an average of 30.95 million fragments per sample and 89.2% uniquely aligned reads per sample (see Supplementary Material). Our final RNA-seq dataset comprised over 13.5 K unique transcripts. Principal components analysis of RNA-seq data for the rotenone-treated and vehicle control rats revealed good separation between the samples comprising the two groups (Fig. 3A). Comparison of expression levels between the rotenone-treated and vehicle groups revealed 345 differentially expressed genes (DEGs) at FDR < 0.05, with 83 significantly upregulated and 262 significantly downregulated, as shown in the volcano plot (Fig. 3B). Considering just the significant (FDR < 0.05) upregulated genes with larger effect sizes, we observed 35 with log_2_FC > 0.5 and 13 with log_2_FC > 1. For the significantly downregulated genes, there were 218 with log_2_FC < -0.5 and 8 with log_2_FC < -1.

The most significant gene (*p* = 3.16 × 10^− 11^, *q* = 7.65 × 10^− 8^) that was upregulated in the rotenone group was the angiopoietin 2 gene (*Angpt2*). This gene is notable because it was also found to be significantly upregulated in post-mortem brains from PD patients compared to controls (Huang et al. 2022). The most significant (*p* = 2.53 × 10^− 19^, *q* = 3.67 × 10^− 15^) downregulated gene in the rotenone group was cytochrome P450 family 51 (*Cyp51*), also known as lanosterol 14α-demethylase. Also among our significant DEGs was the dopa decarboxylase (*Ddc*) gene that was significantly downregulated (*p* = 5.68 × 10^− 5^, *q* = 6.81 × 10^− 3^) in the rotenone-treated group by approximately 30% relative to controls. This gene encodes the dopa decarboxylase enzyme, which is involved in the decarboxylation of L-DOPA to dopamine, a pathway typically disrupted in PD. Complete results for the RNA-seq analysis are provided in **Supplementary Table ****S1**.

### Pathway Analysis of Differentially Expressed Genes

To discover common biological themes among the findings, we conducted Gene Ontology (GO) and pathway analysis on the total set of 345 differentially expressed genes (Table 1). This analysis was in three parts, and we report only top GO/pathway findings significant after correction for multiple testing (FDR < 0.05). In the first part, we analyzed enrichment of GO Biological Processes, for which the most significant GO was “cellular stress response to acid chemical”. This GO included just two genes, both of which were significantly downregulated by rotenone in our data: the Krueppel-like factor 2 (*Klf2*) gene (*p* = 7.9 × 10^− 4^, *q* = 3.7 × 10^− 2^) and the Vascular endothelial growth factor A (*Vegfa*) gene (*p* = 2.0 × 10^− 5^, *q* = 3.5 × 10^− 3^). The second and third ranked GOs, by fold enrichment, were “regulation of lipoprotein particle clearance” and “positive regulation of apoptotic cell clearance” respectively. In the second part, we tested for enrichment of rotenone DEGs in GO Cellular Components. Here, the significant GOs included several pertaining to the extracellular matrix. The top two terms by fold enrichment, “collagen-containing extracellular matrix” and “extracellular matrix”, both included our most significantly up-regulated gene *Angpt2* (see above). Other GOs showed significant enrichment for both the Biological Process and Cellular Component analysis but the majority were nested within, or similar to, the top pathways shown in Table 1. The complete output is shown in **Supplementary Table ****S2**.

**Table 1:** Significant (FDR < 0.05) Gene Ontologies (GOs) and PANTHER pathways, ranked by fold enrichment, among 345 differentially expressed genes in rat striatum following rotenone administration

*GO Biological Process*	*ngenes*	*overlap*	*expected*	*Fold Enrichment*	*P value*	*FDR*
cellular stress response to acid chemical (GO:0097533)	2	2	0.03	68.27	2.14E-04	3.89E-02
regulation of lipoprotein particle clearance (GO:0010986)	6	3	0.09	34.13	6.03E-05	1.98E-02
positive regulation of apoptotic cell clearance (GO:2000427)	8	3	0.12	25.6	1.65E-04	3.28E-02
*GO Cellular Component*	*ngenes*	*overlap*	*expected*	*Fold Enrichment*	*P value*	*FDR*
collagen-containing extracellular matrix (GO:0062023)	192	12	2.81	4.27	2.81E-05	6.92E-03
extracellular matrix (GO:0031012)	410	23	6.01	3.83	4.68E-08	3.07E-05
external encapsulating structure (GO:0030312)	412	23	6.03	3.81	5.11E-08	2.52E-05
*PANTHER Pathways*	*ngenes*	*overlap*	*expected*	*Fold Enrichment*	*P value*	*FDR*
Circadian clock system (P00015)	10	3	0.15	19.39	8.95E-04	4.78E-02

Finally, analysis of our findings in Panther Pathways revealed significant enrichment of rotenone DEGs in the Circadian Clock System. Three circadian clock system genes were significantly differentially expressed in the rotenone-exposed subjects. The cryptochrome circadian regulator 1 (*Cry1*) gene (*p* = 1.97 × 10^− 4^, *q* = 1.5 × 10^− 2^) and the aryl hydrocarbon receptor nuclear translocator-like (*Arntl*) gene (*p* = 3.94 × 10^− 4^, *q* = 2.34 × 10^− 2^) were both significantly upregulated in the rotenone-exposed subjects, while the period circadian regulator 3 (*Per3*) gene was significantly downregulated (*p* = 3.05 × 10^− 4^, *q* = 2.0 × 10^− 2^). Circadian dysregulation is a core feature of PD (Leng et al. 2019; Nassan and Videnovic 2022).

### Targeted Validation of Findings by qPCR

**Fig. 4:**
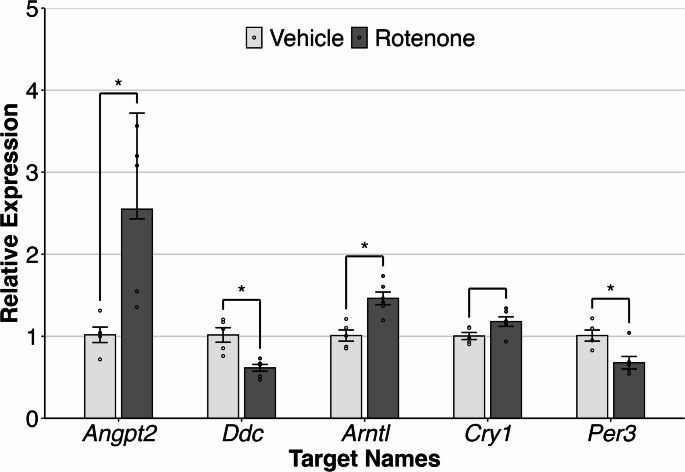
Bar plot of qPCR assays to validate rotenone-induced differential expression of genes in the striatum detected in RNA-seq. Plots show the relative quantification with controls normalized to have a mean expression value of 1 (* = *p* < 0.05). Samples are *n* = 6 rotenone-treated rats and *n* = 5 control rats. Data for all five genes did not deviate significantly from normality using the Shapiro-Wilk test

To validate gene expression findings from RNA-seq using a different technology, we used RT-qPCR. First, we assayed *Ddc* via RT-qPCR because of its importance in dopamine metabolism and PD. We also assayed *Angpt2* because of its status as our most significantly upregulated gene, in addition to it being a top finding in post-mortem PD brain tissue, as mentioned above. Finally, we assayed the three circadian rhythm genes *Arntl*, *Cry1* and *Per3*. The number of biological replicates was the same as the RNA-seq, i.e. striatal RNA from *n* = 6 rotenone-exposed rats and *n* = 5 control rats. Four of the five genes showed significant effects (*p* < 0.05) in the same direction as the RNA-seq study, as shown in Fig. 4. These were *Angpt2*, *Arntl*,* Ddc* and *Per3*. *Cry1* showed a modest effect in the same direction as seen in the RNA-seq, but was not significant in the RT-qPCR assay. Overall, these results indicate disruption to striatal expression of *Angpt2*, *Ddc* and circadian rhythm genes in the rotenone rat model of PD.

## Discussion

In this study, we used a well-established model of PD, the rat rotenone model, to investigate rotenone-induced changes to gene expression in the striatum. Consistent with prior work (Cannon et al. 2009), the rotenone-treated subjects exhibited weight loss and catalepsy. For subjects receiving the full nine-day course of rotenone treatment and controls receiving the same number of vehicle injections, RNA-seq revealed several hundred DEGs. Overall, our results are indicative of substantial disruption to gene expression by rotenone.

Among our significant DEGs was *Ddc*, or dopa decarboxylase, which encodes the enzyme responsible for conversion of L-DOPA to dopamine. L-DOPA, also known as levodopa, has long been used as a treatment for PD (LeWitt and Fahn 2016). A reduction in *Ddc* might lead to reduced L-DOPA in rotenone-treated rats but tyrosine hydroxylase, the enzyme responsible for converting dietary tyrosine to L-DOPA, is typically considered the rate-limiting step in this process. It has been previously reported that rotenone leads to increased dopamine turnover, rather than less (Cannon et al. 2009). A possible explanation for the reduced levels of *Ddc* transcript in rotenone-treated subjects is the loss of dopaminergic neurons that express this gene.

The most significantly upregulated gene in our RNA-seq analysis was *Angpt2*, which encodes the angiopoietin 2 (Ang-2) growth factor. Expression of this gene is tightly controlled and mostly restricted to the vascular endothelium (Scholz et al. 2015). While angiopoietin 1 stabilizes the blood brain barrier, via regulation of vessel maturation though activation of receptor tyrosine kinase 2, Ang-2 is an antagonistic ligand and is stored in endothelial Weibel-Palade bodies from where it can be rapidly released in inflammatory conditions (Fiedler et al. 2006). Ang-2 is thought to induce endothelial permeability, i.e. blood brain barrier leakiness (Lukasz et al. 2017). Notably, a single cell RNA-seq study of post-mortem PD brains found *ANGPT2* to be of the most significantly upregulated transcripts in endothelial cells (Huang et al. 2022). This observation, in conjunction with the known expression patterns of angiopoietin 2, would imply that the *Angpt2* signal in our bulk RNA-seq study is also coming from endothelial cells. The correspondence between a human post-mortem study of PD brain and our study of the rat rotenone model with respect to the upregulation of *Angpt2* expression is encouraging. Given its role in the cerebral vasculature, Ang-2 upregulation may provide a mechanistic link between cerebral vascular permeability abnormalities seen in PD and other neurodegenerative disorders (Gray and Woulfe 2015; Knox et al. 2022). Furthermore, obstructive sleep apnea (OSA) is a sleep disorder seen often in PD (Anderson et al. 2025) and genetic association studies have observed that OSA risk is associated with variants in the *ANGPT2* gene (Mukherjee et al. 2018). *ANGPT2* has been associated specifically with variation in nocturnal oxyhemoglobin saturation level (Wang et al. 2016). Mechanistically, therefore, *ANGPT2* up-regulation could be affecting sleep quality in PD.

Pathway analysis of rotenone DEGs identified the circadian clock system in our study. Three clock system genes, *Arntl*, *Cry1* and *Per3*, were dysregulated in our RNA-seq and two of the three (*Arntl* and *Per3*) were validated via targeted qPCR. Circadian dysfunction is nearly ubiquitous in patients with PD and is thought to be involved in disruption of the sleep–wake cycle, autonomic nervous dysfunction, cognitive impairment, impaired hormonal secretion, and emotional disturbance (Nassan and Videnovic 2022). Evidence from clinical genetic studies has linked variants in several clock system genes such as *PER1* and *CRY1* to PD (Gu et al. 2015; Xiang et al. 2023). Moreover, a single nucleotide polymorphism in *ARNTL* was previously associated with PD risk in a Chinese sample (Gu et al. 2015). A prior rat study of rotenone also detected disruption to circadian genes, albeit using a different experimental procedure. Li et al. (2019) used a single injection of bacterial lipopolysaccharide to elicit neuroinflammation, followed by low dose (0.5 mg/kg) rotenone, five times per week for four weeks. Their study then focused on expression of selected circadian system genes and found downregulation of genes such as *Bmal1*, *Clock* and *Npas2*. However, the specific genes detected in our study (*Arntl* and *Per3*) were not tested in theirs. The brain regions assessed were also different, with our study focusing on the striatum and that of Li et al. focusing on gene expression in the cortex. Nevertheless, the overall theme of disruption to the circadian system by rotenone is consistent and therefore seems worthy of further study in the context of PD using the rat rotenone model.

The *ARNTL* association detected by Gu et al. (2015), mentioned above, was from a candidate gene study of clock genes. To determine if any of our rotenone DEGs overlapped with PD genes from genome-wide association studies (GWAS), we downloaded all current genome-wide significant (*p* < 5 × 10^− 8^) associations with PD from GWAS catalog (Cerezo et al. 2025) and matched the IDs the human genes ascribed to these SNPs to their rat orthologs. We performed a test of enrichment with these genes and our rotenone DEGs and found this to be non-significant (*p* = 0.29). Only five of our genes overlapped with PD GWAS risk genes. These genes are ADAM metallopeptidase with thrombospondin type 1 motif 20 (*Adamts20*), cyclin T2 (*Ccnt2*), polycomb group ring finger 3 (*Pcgf3*), ribosomal protein S6 kinase like 1 (*Rps6kl1*), and stabilin 1 (*Stab1*). These genes serve diverse functions. *Adamts20* encodes a zinc-dependent protease that is secreted into the extracellular matrix and may be involved in tissue remodeling (Nandadasa et al. 2019). *Ccnt2* is a regulatory subunit of the positive transcription elongation factor b (P-TEFb) complex, which is involved in a range of biological activities including cell cycle control, cytokine signaling and oligodendrocyte differentiation (Kuypers et al. 2016). *Pcgf3* encodes a ring finger protein involved in transcriptional control (Almeida et al. 2017), *Rps6kl1* has not been well characterized and *Stab1* encodes a multifunctional scavenger receptor induced during chronic inflammation (Kzhyshkowska 2010). It is conceivable that altered expression of some of these genes in response to rotenone may be as a result of inflammation or other toxic effects, but these genes are also implicated in PD risk. At the moment, there is no discernable thematic similarity that would point to a common mode of action by these genes in PD pathophysiology. Ultimately, the extent to which an overlap should be expected between the biological pathways affected by a PD environmental risk factor (rotenone) and PD genetic risk variants is debatable.

Our study has some limitations. First, we only looked at male rats. A more complete follow-up study should also include females to test for potential sex differences. Second, we only analyzed gene expression in rats that received a full course of rotenone treatment. It may also be informative to look at rats that showed early signs of susceptibility but did not receive all the rotenone doses. Third, gene expression analysis is correlational, not causal. Many DEGs will be secondary effects, downstream of rotenone’s immediate effects. It is possible that many of our findings will not have direct relevance to PD. Fourth, our study was not designed to probe circadian rhythms but we found circadian genes. Our samples were collected within a 2-hr time window each day, which should limit diurnal variability in expression, but future studies should look at a broader range of times across the diurnal cycle. Fifth, gene expression effect sizes for many of the significant genes were not large. Further work is needed to characterize the effects at a more granular level. Single cell gene expression analysis could quantify effects in specific cell types, which would provide a more nuanced view of rotenone’s effects. The results of our study support that rotenone causes substantial changes to gene expression in bulk, thus justifying progression to single cell analysis in future studies.

## Supplementary Information

Below is the link to the electronic supplementary material.

Supplementary Material 1 (PDF 91.1 KB)

Supplementary Table S1 - Complete RNA-seq results

Supplementary Table S2 - Complete Gene Ontology results

## Data Availability

Raw sequence data are available to download from the Sequence Read Archive at the National Center for Biotechnology Information with accession number PRJNA1310653 (https://www.ncbi.nlm.nih.gov/sra/PRJNA1310653).
