# Supplementary material for: Striatal Dysregulation of Angpt2 and Circadian Gene Expression in a Rotenone Rat Model of Parkinson’s Disease
Source: J Mol Neurosci. 2026 Apr 2;76(2):58. doi: 10.1007/s12031-026-02506-z (PMC13046621; doi:10.1007/s12031-026-02506-z)
Supplement: Supplementary file 1 — Supplementary Material 1 (PDF 91.1 KB) [file 12031_2026_2506_MOESM1_ESM.pdf]

Supplementary materials

RNA-seq and data processing

Polyadenylated transcripts were enriched using oligo(dT) and subjected to standard, non-stranded PE150 Illumina RNA sequencing, targeting 30M clusters per sample. The mean number of sequenced fragments per sample was 30.95 million (range 25.5-35.6 million fragments). Raw data as fastq files were analyzed using FastQC v0.11.9 (quality base calls, CG content distribution, duplicate levels, complexity level) (Andrews 2010). Sequencing adapters were removed using TrimGalore v.0.6.4_dev (Krueger et al. 2023). Reads were aligned to the Rn7 rat reference genome using STAR v.2.5.2b (Dobin et al. 2013). Alignment to the rat reference genome was successful with a mean of 89.2% uniquely aligned reads per sample (range 89.39 - 90.74%). Reads were counted on gene level using featureCounts v2.0.1, using the transcript annotation Rattus_norvegicus.mRatBN7.2.108.chr.gtf.

RNA-seq analysis

Principal components analysis was applied to RNA-seq data to evaluate the overall similarity/difference of samples. RNA-seq counts were preprocessed and analyzed for differential expression using edgeR v.3.30.0 (Robinson et al. 2010). P-values for differentially expressed genes were corrected using a False Discovery Rate (FDR) threshold of 5% (Benjamini and Hochberg 1995). Analysis coding and plots for data visualization used R version 4.1.3 in RStudio.

Pathway Analysis

To identify common biological themes among the top findings, these genes were entered into pathway analysis using the online resource at GeneOntology.org (Mi et al. 2017). The organism was specified as *Rattus norvegicus* and we used Gene Ontology (Ashburner et al. 2000; Gene Ontology Consortium et al. 2023) and PANTHER version 18.0, PANTHER Overrepresentation Test (Released 20231017) with FDR correction at 5%.
